# Predictors of COVID-19 Pandemic-Related Pregnancy Stress: Prenatal and Postpartum Experiences in Canada

**DOI:** 10.3390/ijerph22081302

**Published:** 2025-08-20

**Authors:** Sigourney Shaw-Churchill, Karen P. Phillips

**Affiliations:** Interdisciplinary School of Health Sciences, Faculty of Health Sciences, University of Ottawa, Ottawa, ON K1N 6N5, Canada; sshaw051@uottawa.ca

**Keywords:** pregnancy, COVID-19, pandemics, coping behavior, social support, stress, psychological

## Abstract

The COVID-19 pandemic and related public health and hospital restrictions directly influenced Canadian perinatal healthcare. This study aimed to evaluate predictors of pandemic-related pregnancy and postpartum stress in Canada. A sample of 398 women with Canadian pandemic pregnancy experiences completed an online cross-sectional survey between September 2021 and February 2022. Demographic factors, perinatal healthcare characteristics, and psychometric measures including Oslo Social Support Scale (OSSS-3) and Brief COPE were analyzed by independent hierarchical generalized linear models (GLM) to identify predictive variables associated with prenatal and postpartum pandemic-related pregnancy stress scales (PREPS). Respondents reported low social support, low–moderate Problem-Focused and Emotion-Focused Coping scores, with low Avoidant Coping. Middle income and canceled prenatal care appointments were associated with prenatal PREPS-Preparedness Stress, with provider satisfaction negatively associated. Avoidant Coping was positively associated with both prenatal and postpartum Preparedness Stress and Infection Stress scores, whereas Problem-Focused Coping was associated with both prenatal and postpartum Positive Appraisal. High COVID-19 rates and region of healthcare were associated with prenatal and postpartum Infection Stress. Our findings that perinatal healthcare characteristics and psychometric measures, rather than demographic characteristics, were greater predictors of pandemic-related stress reflect the broad societal disruptions that shaped Canadian pregnancy experiences in our sample of mostly high income, well-educated, non-racialized women.

## 1. Introduction

The perinatal transition is associated with adjustments to pregnancy and the anticipation of newborn care, conceptualized as changes in four dimensions—biological, psychological, cognitive, and social [1]. Pregnancy and childbirth experiences are shaped by individual factors such as gravidity and parity, socioeconomic status (SES), and the quality of healthcare including satisfaction with providers [2]. Stress is a typical feature of the perinatal transition, influenced by pregnancy complications, fears of childbirth, lack of social support, fatigue, and competing childcare demands, all of which may impact pregnancy outcomes [3,4,5]. Societal and healthcare disruptions associated with COVID-19 pandemic further exacerbated experiences of perinatal stress [6,7]. Canadian perinatal healthcare is grounded in principles of woman-centered care [8] including autonomy, respect, informed choice, shared decision-making, and empowerment [9,10]. Pandemic-related public health restrictions challenged the quality of perinatal healthcare and therefore pregnancy experiences in Canada due to limitations to perinatal support companions, lack of continuity of care, and reductions in perinatal healthcare services and personnel [11,12,13,14,15,16].

The emergence of COVID-19 in Canada, as in most countries, was accompanied by public health restrictions including lockdowns, travel restrictions, social distancing, masking in public spaces, and mandatory vaccination for many employment sectors and entertainment/sport and restaurant venues [17,18,19,20]. For many Canadians, these public health restrictions intensified the transitional adjustments to pregnancy and newborn care, caregiving and homeschooling of other children, and economic challenges for low SES families [12,14,21,22,23]. Perinatal depression and anxiety among Canadians increased during the pandemic, particularly among women with disruptions to their income, childcare, work–life balance, and prenatal healthcare [13,15,23].

It is evident that societal and healthcare restrictions adversely affected Canadian pandemic pregnancy experiences [12,14,23,24,25,26]. Gaps in this literature include quantification of the individual and interactive influences of demographic, healthcare, and psychosocial factors on pregnancy stress. The Pandemic-Related Pregnancy Stress Scale (PREPS-Prenatal [6,7,27]) was developed using a sample of American pregnant women, with a later version created to assess postpartum stress (PREPS-Postpartum [28]). Studies from multiple European countries [29,30,31,32,33,34,35,36,37,38,39] and the United States [6,7,28] have implemented versions of these scales, evaluating associations of PREPS scores with fear of childbirth [37], neonatal body length [36], anxiety [31,38,39,40], and depression [35]. Canada’s universal healthcare system, distributed by 13 provinces/territories, together with robust public health interventions which culminated in widespread COVID-19 vaccination uptake, provides a distinct context to evaluate pandemic pregnancy stress. Given the significance of the COVID-19 pandemic on social, economic, and health systems in Canada and consequently, experiences of pregnancy, our exploration of the factors that influenced pandemic-related pregnancy and postpartum stress adds to the body of Canadian pandemic literature. 

## 2. Methods

### 2.1. Participants

Participants, aged 20–45 years, with pregnancy-related healthcare experiences in Canada after January 2020, who understood English, were invited to respond to the survey. Participants were purposively recruited via social media channels, community groups and networks, as well as ‘snowball’ sampling from 1 September 2021 to 1 February 2022. This time period corresponds with the emergence of Delta (Fall 2021) and Omicron (Winter 2022) COVID-19 variants [17]. Individuals who received prenatal care or gave birth outside of Canada were excluded. Individuals who had immigrated to Canada within the past five years were excluded from participation due to potential barriers to healthcare access associated with limited social integration.

### 2.2. Data Collection

The online survey (SurveyMonkey^TM^, San Mateo, CA, USA), framed by principles of woman-centered care [9,10] and adapted from “Canadian Maternity Experiences Survey” [2,41], explored COVID-19-related experiences of prenatal care, labor and delivery (L&D) care, psychometric measures of pandemic stress, coping, and social support, and demographics. Survey questions were formatted as multiple choice, open answer, and Likert scales.

Psychometric measures included the Pandemic-Related Pregnancy Stress Scale (PREPS) prenatal [6,7,27] and postpartum [28] versions, the Oslo Social Support Scale (OSSS-3) [42], and the Brief COPE [43,44,45,46]. Respondents’ social support available from neighbors, family, and the community was assessed using the total score of the three-item OSSS-3 [42], with Cronbach’s α = 0.661 in this sample. OSSS-3 scores were interpreted as follows: poor social support (score 5–8), moderate support (score 9–11), and strong support (score 12–14) [42].

The Brief COPE is a shortened version of the full COPE developed by Carver et al. [43] and is a validated measure of coping ability based on 14 two-factor scales, further categorized into 3 distinct coping styles [43,44,45,46]. High Problem-Focused Coping mean scores (active coping, use of instrumental support, positive reframing, planning) are indicative of pragmatic resilience coping skills [44]. Emotion-Focused Coping comprises COPE measures (use of emotional support, venting, humor, acceptance, religion, self-blame), with high mean scores reflecting strategies used to constructively mitigate emotional distress, whereas high mean Avoidant Coping scores (self-distraction, denial, substance use, behavioral disengagement) suggest maladaptive strategies to disengage from the stressful situation [43,44,45,46]. Brief COPE subscales (Problem-Focused Coping Cronbach’s α = 0.801 and Avoidant Coping α = 0.656) demonstrated acceptable internal consistencies. Emotion-Focused Coping was deemed acceptable based on consistency (α = 0.613) and inter-item correlation (mean = 0.126), despite missing one of the venting co-factors (COPE21) which was not collected.

PREPS-Prenatal [6,7,27] and PREPS-Postpartum [28] were developed and validated by Preis et al. between 2020 and 2022, and are measures of pandemic-related stress during prenatal and postpartum periods. Here, a slight modification of the original protocol due to the omission of one item from each scale, 14 questions, each measured as 5-point Likert, were organized into two factors representing different aspects of stress—Preparedness and Infection—and one factor, Positive Appraisal, related to coping strategies. Internal consistency of both PREPS-Prenatal and Postpartum subscales was strong (Cronbach’s α ≥ 0.81), with moderate consistency demonstrated by PREPS-Prenatal Positive Appraisal (α = 0.66, inter-item correlation mean = 0.393). Despite the omission of the factor “I am concerned that people won’t be able to help me care for my baby after birth” from the PREPS-Prenatal Preparedness Stress score [6] and the factor “I am concerned about going to postpartum check-ups” from the PREPS-Postpartum Infection Stress score [28], Cronbach’s α was 0.83 and 0.85, respectively, indicating strong internal consistency and supporting their use as outcome variables in subsequent analyses.

### 2.3. Data Analysis

We received a total of 1062 responses, of which 664 were eliminated from the sample as the respondents did not meet the inclusion criteria for this analysis (not pregnant after January 2020, no completion of the demographics and PREPS-Prenatal questions). Survey data was extracted from Survey Monkey to Microsoft Excel (v. 2504), cleaned to remove incomplete/missing responses, yielding a final sample of 398 women. Respondents were classified as primigravid for first pregnancies during COVID-19, with multigravid used to describe participants with pregnancies both before and after 1 January 2020. As pregnancy experience, rather than live birth, was the emphasis of the study, we have refrained from using the terms ‘primiparous’ and ‘multiparous’. PREPS-Prenatal subscale mean scores, completed by the full sample, and the PREPS-Postpartum subscale mean scores, completed by women who reported childbirth (postpartum respondents) were analyzed as outcome variables. Predictors of PREPS-Prenatal and Postpartum subscales (Preparedness Stress, Infection Stress, Positive Appraisal) were evaluated by a series of Generalized Linear Models (GLM; linear regression) using SPSS Statistics (v. 29/30. IBM Corp. Armonk, NY, USA) using a hierarchical approach (Box 1). Preliminary block analyses (demographics, pregnancy care characteristics, and psychometric scales of social support/coping) were each independently evaluated for goodness-of-fit and significance. Univariate GLM were used to obtain R^2^ and adjusted R^2^ values for the final models, retaining original predictors. Statistically significant variables (*p* < 0.05) identified from each preliminary block model were retained in a final multivariable GLM to assess independent associations.

Box 1 **Potential Predictors of PREPS-Scales.**

**PREPS-Prenatal**

**PREPS-Postpartum**

**Block 1: Demographics**
AgeIndigenous Status (yes, no)Racialized Status (yes, no)Immigrant Status (yes, no)Education (some/highschool, college, university, post-graduate)
**Household Income (<$50,000, $50,000–$79,999; $80,000–$99,999; $100,000–$149,999, >$150,000)**
Block 2: Pregnancy Healthcare Characteristics ^1^Gravidity ^2^ (primigravid, multigravid)Healthcare Provider (OB/GYN, family physician, midwife)Healthcare Provider Satisfaction (good/excellent, very poor/poor/fair)Province of Healthcare ^3^ (West, Prairies, Ontario, Quebec, Atlantic)COVID-19 Conditions in Healthcare Region ^4^ (Hot-Spot, Moderate, Low–No COVID-19)Location of Healthcare  (hospital, clinic/primary care, birthing/community health center/home)Birthplace of Choice (yes, no)Prenatal Education (yes, no)Mode of Delivery (vaginal, C-section)Cancelation Prenatal Appointments (yes, no)Support Companion Restricted (yes, no)Prenatal Appointments Rescheduled as Virtual/Phone (yes, no)
Appointment Support Companion Permitted (yes, no)

**Block 3: Psychometric Scales: Social Support/Coping**
Oslo Social Support Scale (OSSS-3)COPE-Problem-Focused Coping SubscaleCOPE-Emotion-Focused Coping SubscaleCOPE-Avoidant Coping Subscale
**Final Generalized Linear Model—statistically significant variables from Blocks 1, 2, 3**

Notes: ^1^ As highly skewed dichotomous variables, L&D location (hospital-97.7%) and L&D support companion (yes-98.2%) were excluded from analysis. ^2^ Gravidity determined as primigravid—pregnant for the first time during the pandemic, or multigravid—pregnant both before 1 January 2020 and during the pandemic. ^3^ Regions as follows: West (British Columbia, Alberta), Prairies (Manitoba, Saskatchewan), and Atlantic (New Brunswick, Nova Scotia, Prince Edward Island, Newfoundland and Labrador). Canadian territories were not included in the analysis due insufficient sample size. ^4^ COVID-19 conditions in healthcare region were characterized by respondents as High (a COVID-19 hot-spot, frequent or lengthy local lockdowns, regular outbreaks, and restaurants/gyms mostly closed), Moderate (moderate COVID-19 infection rates, occasional local lockdowns, some outbreaks and restaurants/gyms sometimes closed), and Low (low COVID-19 infection rate, lockdown only because of province, infrequent outbreaks, restaurants/gyms mostly open), and No COVID-19 (what pandemic? Life has been pretty normal; no lockdowns, no outbreaks, all businesses open). OB/GYN—obstetrician/gynecologist, L&D—labor and delivery.

### 2.4. Ethics

Participants were given detailed information about the study purpose, risks, and benefits of participation, and were asked to indicate their informed consent prior to commencement of the survey. Upon completion of the survey, participants were given the opportunity to enter a random draw for an Amazon gift card valued at $50 CAD. Ethics approval for this study was obtained from the University of Ottawa Research Ethics board (REB file number H-05-21-6902).

## 3. Results

### 3.1. Demographics

All participants identified as women, such that use of binary language is not meant to exclude other genders who can experience pregnancy. The sample was comprised predominantly of non-racialized Canadians (Table 1), with limited participation from visible minorities (6.5%), Indigenous (4.8%), and immigrant (5.8%) communities. Respondents reported high educational attainment, with many having attained a university or post-graduate degree (63.4%), with high household annual incomes of at least $100,000 (63.4%).

### 3.2. Social Support, Coping

Participants scored an average of 8.21 (SD = 2.8) on the OSSS-3, indicating poor social support (Table 1, Supplementary Table S1). Mean sample Problem-Focused (2.6 ± 0.6) and Emotion-Focused (2.34 ± 0.42) Coping scores suggested that engagement in constructive emotional measures was low to moderate. Maladaptive coping strategies were less frequently employed (mean Avoidant Coping score 1.71 ± 0.4).

### 3.3. Prenatal Care 

Respondents’ prenatal healthcare providers (HCPs) were typically obstetrician/gynecologists (OB/GYNs) or primary care physicians (77.9%), perceived as generally satisfactory (81.2%; Table 2). Prenatal healthcare was accessed in regions perceived to have moderate or high rates of COVID-19 infection (80.9%), and almost half of the sample received healthcare in Ontario (48.2%). Respondents were generally unaccompanied at prenatal appointments (76.9%), with the majority reporting experiences of appointment cancelations (74.4%). Although one-fifth of the sample reported high prenatal Preparedness (18.6%) and Infection (21.9%) Stress, mean sample scores (2.96 ± 1.0, and 3.03 ± 1.04, respectively) were both moderate, with low Positive Appraisal (2.10 ± 0.86). Indigenous, racialized, and immigrant respondents exhibited higher PREPS-Prenatal Stress scores (Supplementary Table S2).

### 3.4. Predictors of Prenatal Pandemic Pregnancy Stress

Potential predictors for each of the PREPS-Prenatal subscales—Preparedness Stress, Infection Stress and Positive Appraisal- were evaluated by GLM using a hierarchical strategy as described (Box 1). Preliminary GLM (demographics, prenatal characteristics, social support/coping styles) demonstrated acceptable model fits (deviance/df = 0.7–1.0) and were statistically significant based on likelihood ratio chi-square tests (*p* < 0.05), with the exception of the model evaluating associations between prenatal characteristics and Positive Appraisal, which was not significant (*p* > 0.05; Supplementary Tables S3–S11). Approximately 20% of the variance in Preparedness Stress and Infection Stress was explained by the included predictors for each model, whereas Positive Appraisal was minimal. Statistically significant variables identified in these preliminary models (*p* < 0.05) were retained and integrated in the final multivariable models for each outcome variable (PREPS subscales).

Demographic factors (income, age), prenatal healthcare characteristics (canceled appointments, HCP satisfaction, prenatal region COVID-19 rates), and psychometric variables (OSSS-3, Brief COPE-Avoidant Coping subscale) significantly associated with PREPS-Preparedness Stress in the preliminary GLM analysis (Supplementary Tables S3–S5) were integrated into the final model (Table 3). Income between $50,000 and $79,999 (reference: >$150,000; B = 0.49; 95% CI: [0.15, 0.83], *p* < 0.01), Avoidant Coping (B = 0.76, CI: [0.52, 1.0], *p* < 0.001), experience of canceled prenatal appointments (B = 0.52, CI: [0.30, 0.74], *p* < 0.001), and high COVID-19 rates in prenatal care region (B = 0.34, CI: [0.07, 0.62], *p* = 0.014) were positively associated with Preparedness Stress, whereas HCP satisfaction was negatively associated (B= −0.34, CI: [−0.60, −0.08], *p* = 0.011).

Statistically significant variables identified from the preliminary GLM block analysis—Indigenous status, Avoidant Coping, prenatal appointment support companion permitted, canceled prenatal appointments, prenatal care region, and local COVID-19 rates (Supplementary Tables S6–S8)—were integrated into the final PREPS-Infection Stress model (Table 3). Avoidant Coping (B = 0.40, CI:[0.14, 0.66], *p* = 0.002) and both high (B = 0.58, CI:[0.27,0.89], *p* < 0.001) and moderate (B = 0.35, CI:[0.06, 0.63], *p* = 0.017) COVID-19 rates in prenatal care regions were associated with Infection Stress. In contrast, receiving prenatal care in a Canadian prairie province (reference: Atlantic provinces) was negatively associated with Infection Stress (B = −0.47, CI: [−0.83, −0.11], *p* = 0.011). 

Preliminary PREPS-Positive Appraisal GLM were significant for the demographic hierarchical block (*p* = 0.035) and the stress and coping psychometric measures block (*p* < 0.001; Supplementary Tables S9–S11). As the preliminary GLM for prenatal characteristics was not significant (*p* = 0.63), none of these variables were included in the final model. Significant variables from the two retained models–Indigenous status (B = 0.43; CI:[0.31, 0.83], *p* = 0.035), and Problem-Focused Coping (B = 0.40; CI: [0.24, 0.55], *p* < 0.001) were both positively associated with Positive Appraisal in the final model (Table 3).

### 3.5. Labor and Delivery—Sample Characteristics

Demographic, social support, and coping characteristics of the subset of respondents (*n* = 224) who reported L&D experiences, referred here to as ‘postpartum respondents’, were similar to the main sample (Table 4), although racialized and immigrant respondents exhibited lower social support (Supplementary Table S12). Most of these respondents were primigravid during the pandemic (58%), received L&D care from either an OB/GYN or a primary care physician (86.2%) and were satisfied (93.3%) with their healthcare teams (Table 5). Consistent with experiences of prenatal healthcare, the majority of participants reported L&D care in regions characterized by moderate–high COVID-19 rates (81.7%). Respondents most commonly gave birth by vaginal delivery (66.1%) in hospital (84.6%)—their choice of birthplace (89.7%), whereas most respondents were accompanied by a L&D support companion (98.2%); widespread experiences of restrictions to support companions (93.8%) were reported. Postpartum PREPS-Preparedness Stress (3.15 ± 1.0) was slightly higher in this postpartum subsample compared to the full sample mean prenatal score (2.96 ± 1.0), with immigrant respondents’ postpartum score even lower (2.59 ± 1.09, Supplementary Table S13). A larger proportion of postpartum respondents reported high Preparedness Stress (score ≥ 4.0, 22.3% vs. prenatal full sample: 18.6%, Table 2). PREPS-Infection Stress mean scores were identical across both prenatal and postpartum measures (3.03), with 22.8% reporting high postpartum Infection Stress. Indigenous respondents scored higher (3.43), whereas immigrant respondents demonstrated lower postpartum Infection Stress (2.39; Supplementary Table S13) Postpartum respondents PREPS-Positive Appraisal scores (2.6 ± 1.07) were slightly higher compared with the full sample prenatal PREPS-Positive Appraisal mean score (2.10 ± 0.86).

### 3.6. Predictors of Postpartum Pandemic Pregnancy Stress

As described, a hierarchical approach evaluated demographic factors, L&D characteristics, and psychometric measures of social support and coping in individual preliminary GLM analyses (Supplementary Tables S14–S22), restricted to participants who reported childbirth (vaginal/C-section, Table 4 and Table 5). These preliminary models (demographic factors, L&D characteristics, social support/coping) exhibited acceptable model fits based on the deviance/df values (0.8–1.1). Slightly less than 20% of the variance in Preparedness Stress and Infection Stress was explained by the included predictors for each model, whereas Positive Appraisal was minimal. For postpartum PREPS-Preparedness Stress, the final model incorporated L&D care and psychometric measures of social support and coping variables identified from statistically significant preliminary GLM (Supplementary Tables S14–S16). The demographics GLM was not significant (*p* = 0.45), and therefore, these variables were excluded. Primigravid status (reference: multigravid) was negatively associated with postpartum PREPS-Preparedness Stress (B= −0.30, CI: [−0.56, −0.046], *p* = 0.02), whereas Avoidant Coping was positively associated (B = 1.09, CI:[0.74, 1.44], *p* < 0.001) in the final model (Table 6).

Preliminary GLMs identified immigrant status, L&D care regions, local COVID-19 rates, and Avoidant Coping (Supplementary Tables S17–S19) as significantly associated with postpartum PREPS-Infection Stress and were thus retained in the final model. High rates of COVID-19 in L&D care regions (B = 0.70, CI: [0.25, 1.1], *p* < 0.001) and Avoidant Coping (B = 0.55, CI:[0.15, 0.94], *p* = 0.01) were positively associated with postpartum Infection Stress. In contrast, L&D care in a western (B = −0.62, CI: [−1.22, −0.02], *p* = 0.04) or a prairie province (B = −0.69, CI: [−1.21, −0.16], *p* = 0.01) were each negatively associated with Infection Stress.

Predictors of postpartum PREPS-Positive Appraisal were based on two preliminary GLM (L&D care and psychometric measures of social support/coping (Supplementary Tables S20–S22). As the demographics GLM was not statistically significant based on likelihood ratio χ^2^ test (*p* = 0.14), these variables were not retained in the final model. Only Problem-Focused Coping was positively associated with postpartum Positive Appraisal (B = 0.46 CI: [0.2, 0.7], *p* < 0.001; Table 6).

## 4. Discussion

Pandemic-related stress was influenced by healthcare and psychosocial factors, rather than demographic characteristics, in our study of Canadian pandemic-related pregnancy stress. Our sample reported moderate Preparedness and Infection Stress, as measured by PREPS [6,7,27], with low social support and relatively low to moderate beneficial coping strategies. Middle income disrupted prenatal healthcare and high regional COVID-19 rates influenced Preparedness Stress, which was mitigated by perceived healthcare provider satisfaction. PREPS-Infection Stress was associated with healthcare region and local COVID-19 infection rates. Avoidant Coping was associated with both prenatal and postpartum measures of Preparedness and Infection Stress. Across all final GLM, individual effect sizes were generally modest, reflecting both the limited range of the PREPS measures and the complex, multilevel influences on pregnancy stress.

### 4.1. Pandemic-Related Pregnancy Preparedness Stress

Pregnancy progression is accompanied by preparations and plans for L&D and newborn care, but typically also includes shopping for baby essentials, and celebratory events such as baby showers [24]. Most of these activities were prevented by pandemic-related healthcare and societal disruptions, which together with reduced social support and isolation, adversely impacted pregnancy experiences [11,12,13,14,47]. Prenatal PREPS-Preparedness Stress scores for our sample were lower than scores reported for Spain [29], Poland [30] and the United States [27,48,49], but were similar or slightly higher than scores from Italy [31,38], Germany [34], Greece [33], and Switzerland [49]. These PREPS score variations may be explained by demographic factors (age, pregnancy complications) and societal differences including access to publicly subsidized perinatal healthcare, and different public health strategies such as lockdown and social isolation policies. Although Canada and the United States share many cultural and lifestyle values, Canada’s approach to the pandemic was very different, shaped by relatively strong uptake of public health measures including COVID-19 vaccination [17] and supported by the existing social, economic, and legal infrastructure, which provides Canadians with universal healthcare and generous parental leaves [50]. Canadian pandemic-specific policies decreased the threshold of insurable employment hours required for pregnant workers to qualify for parental leave benefits, and introduced CERB—Canada Emergency Response Benefit—which provided income support during the pandemic in anticipation of disruptions to the labor market [50]. Beyond these factors, our samples’ lower prenatal PREPS-Preparedness Stress score compared to the American studies is probably best explained by high SES—over 63% reporting household incomes exceeding $100,000—which is not representative of most Canadians. Indeed, respondents with middle incomes ($50,000–$79,999) experienced greater Preparedness Stress, relative to those reporting household incomes over $150,000, whereas age was inversely associated. Our findings are widely supported by the literature, which identifies income security, age, previous pregnancy and childbirth experiences, cognitive capacity to understand health information, and access to social support as key factors in mitigating perinatal stress [1,51,52].

PREPS-Preparedness Stress was slightly higher among our postpartum respondents, compared with the prenatal PREPS scores of the main sample. This may be explained by the challenges of newborn care in the context of low social support and ongoing societal disruptions due to the pandemic [52]. The absence of instrumental social support, including childcare, practical assistance with domestic responsibilities, and opportunities for self-care, may exacerbate emotional and physiological stressors producing fatigue and depressive symptoms [1]. Although gravidity was not a significant predictor of prenatal Preparedness Stress, primigravid respondents reported lower postpartum Preparedness Stress compared to multigravid respondents, in contrast with the experiences of American [28] and Greek [33] women. Parity influences mental and physical health aspects of pregnancy, birth, and postpartum experiences [15,53]. Even before the pandemic, it was well established that multiparous women identify competing childcare obligations as barriers to prenatal care [54,55]. With schools closed during the pandemic, the responsibilities of both childcare and homeschooling shifted to parents [23]—a scenario anticipated by Canadian healthcare workers in the context of the 2003 SARS outbreak [56]. Women disproportionately experience work–family conflict [56,57], which together with pandemic-related income disruptions contributed to increased depression and anxiety symptoms [23].

### 4.2. Pandemic-Related Pregnancy Infection Stress

COVID-19 infection rates and political–public health responses to the pandemic varied globally, producing significant regional variation in prenatal PREPS-Infection Stress scores [49]. Our mean sample prenatal PREPS-Infection Stress score (3.03) ranks in the middle of studies reporting PREPS data. Higher Infection Stress scores were reported in studies from Spain [29], Poland [30,58] and the United States [27,48,49], while lower scores were observed in populations from Italy [31,38], Germany [34], and Switzerland [49]. We have previously reported that the majority of this sample was COVID-19 vaccinated (87%), with very low history of COVID-19 infection (9%) [59], which together with Canada’s robust public health measures [17,18,19] may explain our sample’s relatively moderate perinatal PREPS-Infection Stress score compared with other countries.

However, even within Canada, regional public health measures such as lockdowns, curfews, and mandatory public masking varied considerably [17,18,19]. Notably, the Atlantic Bubble, comprising New Brunswick, Nova Scotia, Prince Edward Island, and Newfoundland and Labrador, represented a travel-restricted region for all non-maritime residents [19]. It was therefore somewhat surprising that PREPS-Infection Stress was reduced for respondents who received prenatal and L&D healthcare in Prairie provinces, and among respondents receiving L&D healthcare in Western provinces (British Columbia and Alberta). This may be explained by the relatively low regional population density of the Western and Prairie provinces [60], which may reflect greater access to outdoor space—a factor consistently reported as protective against perinatal Infection Stress [6,30,49]. This finding may also be related to fewer public health restrictions (Alberta, Manitoba, Saskatchewan) despite significant COVID-19 outbreaks in these regions in August 2021 [17].

Perceptions of high healthcare regional COVID-19 infection rates were independently predictive of prenatal PREPS- Preparedness Stress, and both prenatal and postpartum PREPS- Infection Stress. This is consistent with American [6,27] and Polish [30] studies in which COVID-19 exposure and perceived infection risk are associated with perinatal PREPS-Infection Stress. It is now well-established that SARS-CoV-2 infection during pregnancy is associated with increased maternal morbidity, mortality, and adverse pregnancy outcomes [61,62]; however, significant misinformation related to fertility and other reproductive health outcomes associated with COVID-19 vaccination complicated health promotion efforts and represent challenges for future public health campaigns [63].

### 4.3. Disrupted Perinatal Healthcare

The core principles of woman-centered care include informed choice, autonomy, access to support companions, and continuity of care [9,10]. It is well-established that the pandemic disrupted the quality of perinatal care, particularly childbirth experiences [11,14,15,25], confirmed by our respondents who reported having appointments canceled and rescheduled as virtual visits, and restrictions to support companions. COVID-19 screening protocols, visitor restrictions, and limitations to one L&D support companion admitted only upon active labor were among the pandemic-related changes to Canadian hospital policies [11,12,25,64]. Canadian prenatal patients also reported last-minute HCP changes due to staff shortages or redeployments, disrupting healthcare experiences [14]. Canceled prenatal appointments increased our respondents’ prenatal PREPS-Preparedness Stress, consistent with an American study [27], with HCP satisfaction mitigating this stress. Prior to the pandemic, a systematic review reported that childbirth experiences are greatly influenced by satisfaction with the healthcare quality, with poor care associated with stress and anxiety [52]. Although not significant when adjusted for demographic and psychosocial variables, in our preliminary model of prenatal healthcare characteristics, prenatal appointment cancelation was associated with Infection Stress and attenuated among respondents whose partners were permitted to attend appointments. Even though almost all respondents had a L&D support companion, widespread restrictions to support companions were reported which decreased postpartum-PREPS Positive Appraisal. As described, hospital policies did not permit more than one support companion [11,12,25,64], restricting L&D access to doulas, other children, or family members. Despite disruptions to perinatal healthcare, most of our respondents were satisfied with their prenatal and L&D healthcare providers and location of birth; however, this does not reflect all Canadian perinatal experiences [11,12,14,15,25]. Our findings are supported by the literature [11,25,52,65] that HCP quality and access to support companion of choice helps to mitigate pandemic pregnancy stress. Public health and healthcare institutional pandemic planning should incorporate strategies to ensure patient autonomy, access to perinatal support companions, and adherence to principles of shared decision-making to optimize perinatal experiences.

### 4.4. Special Populations

Even before the emergence of COVID-19, Canadian pandemic planning strategies recommended prioritization of high risk populations typically susceptible to infectious disease burden due to pregnancy, remote geographies, colonization, systemic exclusion, and racism [57,66,67]. Indigenous status initially predicted prenatal PREPS-Infection Stress in our preliminary model; however, this association was no longer significant after adjusting for prenatal healthcare and coping factors in the final model. Indigenous status was, however, significantly associated with prenatal Positive Appraisal, which may reflect resilience against Canada’s legacy of colonialism, cultural genocide, and systemic discrimination [68]. Canadian immigrant status—another pandemic risk factor [57,66,67]—was initially related to lower postpartum Infection Stress in the preliminary model, but this association did not persist after adjusting for L&D region and COVID-19 rates in the final model. Our sample of immigrants reported moderate to high household incomes in excess of $80,000, with most having completed post-secondary school—SES factors which may have mitigated postpartum PREPS-Infection Stress. The high SES of these participants is certainly not reflective of the immigrant population in Canada and may have resulted from our study inclusion criteria that required participants to have lived in Canada for at least 5 years to ensure social inclusion and familiarity with Canada’s healthcare system. Further research is required to better understand the experiences of populations at risk during public health disasters due to the intersections of pregnancy and childbirth with Indigeneity, race/ethnicity, SES, and other factors which may contribute to social exclusion and health disparities [57]. Healthcare providers should explore individual barriers to healthcare literacy, access to services, and immunizations with their patients to help mitigate pandemic-related perinatal stress. 

### 4.5. Social Support and Coping

Social support and coping strategies are inextricably linked. As described, access to support companions throughout perinatal care is a key tenet of woman-centered care [9,10], engages partners in pregnancy and birth [25], and can provide essential L&D patient advocacy [69]. Social support—networks of interpersonal relationships that provide emotional, informational, and instrumental support [42]—protect and mitigate psychological vulnerabilities associated with the perinatal transition [1,52]. Perinatal social support is also an important aspect of satisfaction with healthcare and childbirth experiences, and protects against postpartum depression [70,71,72,73]. Our respondents reported low social support, which was associated only with prenatal Preparedness Stress in preliminary models but not when adjusted for other factors. During the pandemic, social support was limited by public health measures including lockdowns, social distancing, and isolation [18,19], resulting in rising levels of psychological distress [23,74]. Social isolation was further associated with both dysfunctional coping and problem-focused coping [75].

Coping responses encompass a breadth of behaviors—adaptive and maladaptive—that enable individuals to manage their emotions and actions during stressful events [43,76]. Maladaptive behaviors, or avoidant coping, are strategies to disengage from stressors, and are generally associated with postpartum depression and adverse pregnancy outcomes [76]. Our findings are consistent with these studies, as Avoidant Coping was a significant predictor for prenatal and postpartum Preparedness and Infection Stress, although sample mean scores indicated it was not commonly employed as a coping mechanism. Changes to routines, social isolation, and economic and childcare burdens due to the pandemic altered coping strategies in pregnant Canadians [21,26,75]. Avoidant or dysfunctional coping behaviors mediate the indirect associations of negative pandemic experiences such as social isolation, financial stress, and uncertainty, with adverse mental health in pregnancy [75]. In contrast, Positive Appraisal—constructive reframing of stressful events and focus on affirmative events, such as pregnancy and the new baby—is associated with less mental distress [76,77]. Pandemic assessments of pregnancy-related Positive Appraisal report positive associations with measures of personal growth and negative associations with fear of childbirth in American [27] and German [34] samples. Consistent with several PREPS-studies [28,29,33,34,38], we report low to moderate prenatal and postpartum Positive Appraisal scores. Problem-Focused Coping was significantly associated with prenatal and postpartum Positive Appraisal in our sample, suggesting that for some respondents this was an adaptive coping strategy across the perinatal period, as discussed for Indigenous respondents. Given that Problem-Focused Coping can both predict and mitigate pregnancy distress [76,77], its role in mediating the relationship between perinatal stress and Positive Appraisal warrants further study.

### 4.6. Limitations

This study strengthens our understanding of pandemic pregnancy experiences in Canada and the factors influencing perinatal stress. We do note several limitations to our study. First, we used a non-random, purposive sampling strategy, and as such, our findings cannot be generalized to the Canadian population. Our purposive social media sampling introduced selection bias and produced a digitally connected sample which lacked gender, racial/ethnic, and SES diversity, such that our measures of social support, coping, and perinatal stress may underestimate the experiences of many Canadians during the pandemic. Our findings are temporally contextualized by the study period marked by Delata and Omicron variants and widespread availability of COVID-19 vaccines. The multijurisdictional and geographic diversity of the sample adds complexity to the interpretation of the data given that Canadian healthcare, including regional public health, is governed by each province and territory. Although the internal consistency of our psychometric and PREPS measures was acceptable, one item was omitted from the Brief COPE and two of the PREPS subscales, which limits comparability with other studies. Reflecting the lack of universal method for constructing Brief COPE subscales, our approach may differ compared with some of the pregnancy-related literature. Notably, we used mean scores rather than total scores for each coping subscale, which enabled us to align scoring despite the missing Venting item. Consequently, comparisons with other studies should be interpreted with caution. Even though we used a consistent significance threshold (*p* < 0.05), the hierarchical GLM approach involved multiple comparisons, which may have increased the likelihood of Type 1 errors. To support model parsimony, only variables that were statistically significant in the individual models were retained in final GLM. Finally, given the low R^2^ values for our final models, Positive Appraisal in this context is likely explained by additional factors not captured by our study variables.

## 5. Conclusions

We report here that our sample of women who received pregnancy care in Canada demonstrated low to moderate prenatal and postpartum stress, perhaps mitigated by their relatively high socioeconomic status. Respondents indicated low social support and low to moderate Emotion-Focused and Problem-Focused Coping, the latter associated with both prenatal and postpartum PREPS-Positive Appraisal. Although the sample demonstrated low Avoidant Coping, this strategy was associated with both prenatal and postpartum PREPS-Preparedness and Infection Stress. Avoidant Coping, together with perinatal healthcare experiences, including local COVID-19 infection rates, geographical region, and healthcare restrictions, were more likely to be associated with perinatal pandemic stress rather than demographic factors. Further research is needed to measure perinatal pandemic stress in high-risk populations, including Indigenous, immigrant, and racialized communities in Canada. The PREPS instruments are valuable tools to measure prenatal and postpartum stress during the pandemic. Our findings contribute valuable insights to the impacts of broad societal and healthcare disruptions on pregnancy experiences in Canada, adding to the existing literature in this field.

## Figures and Tables

**Table 1:** Demographics.

Characteristic	*n*	%
Age (mean, SD)	32.84 ± 4.0	
Indigenous Status	19	4.8
Racialized Status	26	6.5
Immigrant Status	23	5.8
Education		
Some Highschool/Highschool	35	8.8
College	111	27.9
University	163	41.0
Post-Graduate	89	22.4
Household Income (CAD)		
<$50,000	22	5.5
$50,000–$79,999	40	10.1
$80,000–$99,999	60	15.1
$100,000–$149,999	132	33.2
>$150,000	120	30.2
Psychometric Scales (mean ± SD)		
Oslo Social Support (OSSS-3) Scale	8.21 ± 2.8	
Brief COPE	60.2 ± 9.3	
Problem-Focused Coping	2.6 ± 0.6	
Emotion-Focused Coping	2.34 ± 0.42	
Avoidant Coping	1.71 ± 0.41	

SD—standard deviation.

**Table 2:** Prenatal Care.

Characteristic	*n*	%
Gravidity		
Primigravid	240	60.3
Multigravid	158	39.7
Prenatal Healthcare Provider		
OB/GYN	211	53.0
Family Physician	99	24.9
Midwife	80	20.1
Prenatal Healthcare Provider Satisfaction		
Good/Excellent	323	81.2
Very Poor/Poor/Fair	72	18.1
Province of Prenatal Healthcare ^1^		
West	43	10.8
Prairies	54	13.6
Ontario	192	48.2
Quebec	26	6.5
Atlantic	75	18.8
COVID-19 Conditions—Prenatal Healthcare Region ^1^		
Hot-Spot	146	36.7
Moderate	176	44.2
Low–No COVID-19	73	18.3
Location of Prenatal Healthcare		
Hospital	76	19.1
Clinic/Primary Care	290	72.9
Birth/Community Center/Home	28	7.0
Prenatal Healthcare Appointments Canceled		
Yes	99	24.9
No	296	74.4
Prenatal Healthcare Appointments Rescheduled as Virtual/Phone		
Yes	168	42.2
No	227	57
Partner Could Attend Prenatal Healthcare		
Yes	88	22.1
No	306	76.9
Prenatal Education		
Yes	134	33.7
No	245	61.6
Pandemic-Related Pregnancy Stress Scale (PREPS-Prenatal)		
Preparedness Stress (mean SD)	3.0 ± 1.0	
High Preparedness Stress (≥4.0)	74	18.6
Infection Stress (mean SD)	3.0 ± 1.0	
High Infection Stress (≥4.0)	87	21.9
Positive Appraisal (mean SD)	2.1 ± 0.9	

^1^ Please see Box 1 for healthcare region groups and COVID-19 rate definitions. SD—standard deviation, OB/GYN—obstetrician/gynecologist.

**Table 3:** Prenatal Pandemic-Related Pregnancy Stress scales—PREPS.

Variables	B	Lower CI	Upper CI	*p*-Value
Preparedness Stress ^1^				
Age	−0.01	−0.04	0.01	0.30
Income <$50,000 (>$150,000)	0.33	−0.12	0.78	0.15
Income $50,000–$79,999 (>$150,000) *	0.49	0.15	0.83	0.01
Income $80,000–$99,999 (>$150,000)	0.24	−0.05	0.54	0.11
Income $100,000–$149,999 (>$150,000)	0.14	−0.09	0.38	0.22
OSSS-3	−0.01	−0.05	0.02	0.42
Avoidant Coping ***	0.76	0.52	1.00	<0.001
Prenatal Appointments Canceled (not canceled) ***	0.52	0.30	0.74	<0.001
Satisfied Prenatal Healthcare Provider (not satisfied) *	−0.34	−0.60	−0.08	0.01
COVID-19 Hot-Spot (low–no COVID-19) *	0.34	0.07	0.62	0.01
COVID-19 Moderate (low–no COVID-19)	0.23	−0.03	0.49	0.08
Infection Stress ^2^				
Indigenous (not Indigenous)	0.38	−0.11	0.87	0.13
Avoidant Coping ***	0.40	0.14	0.66	<0.001
Prenatal Healthcare Province—West (Atlantic)	−0.17	−0.57	0.23	0.41
Prenatal Healthcare Province—Prairies (Atlantic) *	−0.47	−0.83	−0.11	0.01
Prenatal Healthcare Province—Ontario (Atlantic)	−0.22	−0.51	0.07	0.13
Prenatal Healthcare Province—Quebec (Atlantic)	0.06	−0.45	0.57	0.82
COVID-19 Hot-Spot (low–no COVID-19) ***	0.58	0.27	0.89	<0.001
COVID-19 Moderate (low–no COVID-19) *	0.35	0.06	0.63	0.02
Appointment Support Companion Permitted (not permitted)	−0.24	−0.50	0.01	0.06
Prenatal Appointments Canceled (not canceled)	0.08	−0.16	0.33	0.50
Positive Appraisal ^3^				
Indigenous (not Indigenous) *	0.43	0.03	0.83	0.03
Problem-Focused Coping ***	0.40	0.24	0.55	<0.001

^1^ Preparedness Stress model goodness-of-fit: deviance/df = 0.76 (df = 312); likelihood ratio χ^2^(11) = 102.2; *p* < 0.001. R^2^ = 0.256; adjusted R^2^ = 0.122. ^2^ Infection Stress model: deviance/df = 0.97 (df = 327); likelihood ratio χ^2^(10) = 40.56; *p* < 0.001. R^2^ = 0.206; adjusted R^2^ = 0.053. ^3^ Positive Appraisal model: deviance/df = 0.67 (df = 340); likelihood ratio χ^2^(2) = 31.60; *p* < 0.001. R^2^ = 0.023; adjusted R^2^ = 0.017 * *p* < 0.05; *** *p* < 0.001. Reference categories are shown in brackets. CI—95% confidence interval.

**Table 4:** Characteristics of postpartum respondents.

Characteristic	*n*	%
Age (mean, SD)	33.3 ± 3.9	
Indigenous	12	5.4
Racialized	14	6.3
Immigrant	10	4.5
Education		
Some Highschool/Highschool	14	6.3
College	61	27.2
University	96	42.9
Post-Graduate Studies	53	23.7
Income		
<$50,000	12	5.4
$50,000–79,999	19	8.5
$80,000-$99,999	33	14.7
$100,000–149,999	77	34.4
>$150,000	70	31.3
Psychometric Scales (mean, SD)		
Oslo Social Support (OSSS-3)	8.4 ± 2.8	
Problem-Focused Coping	2.7 ± 0.6	
Emotion-Focused Coping	2.3 ± 0.4	
Avoidant Coping	1.7 ± 0.4	

SD—standard deviation.

**Table 5:** Labor and delivery characteristics.

Characteristic	*n*	%
Gravidity		
Primigravid	130	58.0
Multigravid	94	42.0
L&D Provider		
OB/GYN	173	78.3
Family Physician	20	9.0
Midwife	28	12.7
L&D Provider Satisfaction		
Good/Excellent	209	93.7
Very Poor/Poor/Fair	14	6.3
Province of L&D Healthcare ^1^		
West	22	10.1
Prairies	33	15.2
Ontario	108	49.8
Quebec	16	7.4
Atlantic	38	17.5
COVID-19 Conditions—L&D Healthcare Region ^1^		
Hot Spot	91	40.8
Moderate	92	41.3
Low-No COVID-19	40	17.9
Location of L&D Healthcare/Birthplace		
Hospital	212	97.7
Birthing Center/Home	5	2.3
Birthplace of Choice		
Yes	201	90.5
No	21	9.5
Mode of Delivery		
Vaginal	148	67.3
C-section	72	32.7
Support Companion		
Yes	220	98.2
No	4	1.8
Support Companion Restricted		
Yes	210	94.2
No	13	5.8
Pandemic Related Pregnancy Stress Scale (PREPS-Postpartum)		
Preparedness Stress (mean SD)	3.15 ± 1.0	
High Preparedness Stress (≥4.0)	50	22.3
Infection Stress (mean SD)	3.0 ± 1.1	
High Infection Stress (≥4.0)	51	22.8
Positive Appraisal (mean SD)	2.6 ± 1.1	

^1^ Please see Box 1 for healthcare region groups and COVID-19 rate definitions. SD—standard deviation, OB/GYN—obstetrician/gynecologist, and L&D—labor and delivery.

**Table 6:** Postpartum pandemic-related stress.

Postpartum PREPS	B	Lower CI	Upper CI	*p*-value
Preparedness Stress ^1^				
Primigravid (multigravid) *	−0.30	−0.56	−0.05	0.02
Avoidant Coping ***	1.09	0.74	1.44	<0.001
Infection Stress ^2^				
Avoidant Coping *	0.55	0.15	0.94	0.01
Immigrant (Canadian)	−0.55	−1.27	0.17	0.13
L&D Healthcare Province—West (Atlantic) *	−0.62	−1.22	−0.02	0.04
L&D Healthcare Province—Prairies (Atlantic) *	−0.69	−1.21	−0.16	0.01
L&D Healthcare Province—Ontario (Atlantic)	−0.24	−0.65	0.18	0.26
L&D Healthcare Province—Quebec (Atlantic)	0.06	−0.62	0.75	0.85
COVID-19 Hot-Spot (low–no COVID-19) ***	0.69	0.25	1.13	<0.001
COVID-19 Moderate (low–no COVID-19)	0.36	−0.07	0.79	0.10
Positive Appraisal ^3^				
Problem-Focused Coping ***	0.46	0.20	0.71	<0.001
Restricted Support Companion (not restricted)	−0.57	−1.22	0.09	0.09

^1^ Preparedness Stress model goodness-of-fit: deviance/df = 0.80 (df = 186), likelihood ratio χ^2^(2) = 38.37, *p* < 0.001. R^2^ = 0.184, adjusted R^2^ = 0.175. ^2^ Infection Stress model: deviance/df = 1.03 (df = 175), likelihood ratio χ^2^(8) = 29.52, *p* < 0.001. R^2^ = 0.197, adjusted R^2^ = 0.098. ^3^ Positive Appraisal model: deviance/df = 1.06 (df = 186); likelihood ratio χ^2^(2) = 15.60, *p* < 0.001. R^2^ = 0.079, adjusted R^2^ = 0.069. * *p* < 0.05; *** *p* < 0.001. Reference categories are shown in brackets. CI—95% confidence interval.

## Data Availability

The data that support the findings of this study are available as Supplementary Tables, with additional data tables related to the broader survey are available as part of S.S.-C.’s MSc thesis: https://ruor.uottawa.ca/items/558c3cde-0556-4109-a43e-15e9068812ab (accessed on 12 August 2025).

## Supplementary Materials

The following supporting information can be downloaded at: https://www.mdpi.com/article/10.3390/ijerph22081302/s1. Table S1. Mean Prenatal Psychometric Scales Scores by Respondent Characteristics, Table S2. Mean Prenatal PREPS Scores by Respondent Characteristics, Table S3. Preliminary GLM: Demographics and Prenatal PREPS-Preparedness Stress, Table S4. Preliminary GLM: Healthcare Characteristics and Prenatal PREPS-Preparedness Stress, Table S5. Preliminary GLM: Social Support, Coping and Prenatal PREPS-Preparedness Stress, Table S6. Preliminary GLM: Demographics and Prenatal PREPS-Infection Stress, Table S7. Preliminary GLM: Healthcare Characteristics and Prenatal PREPS -Infection Stress, Table S8. Preliminary GLM: Social Support, Coping and Prenatal PREPS-Infection Stress, Table S9. Preliminary GLM: Demographics and Prenatal PREPS-Positive Appraisal, Table S10. Preliminary GLM: Healthcare Characteristics and Prenatal PREPS -Positive Appraisal, Table S11. Preliminary GLM: Social Support, Coping and Prenatal PREPS-Positive Appraisal, Table S12. Mean Postpartum Psychometric Scales Scores by Respondent Characteristics, Table S13. Mean Postpartum PREPS Scores by Respondent Characteristics, Table S14. Preliminary GLM: Demographics and Postpartum PREPS-Preparedness Stress, Table S15. Preliminary GLM: Healthcare Characteristics and Postpartum PREPS -Preparedness Stress, Table S16. Preliminary GLM: Social Support, Coping and Postpartum PREPS-Preparedness Stress, Table S17. Preliminary GLM: Demographics and Postpartum PREPS-Infection Stress, Table S18. Preliminary GLM: Healthcare Characteristics and Postpartum PREPS -Infection Stress, Table S19. Preliminary GLM: Social Support, Coping and Postpartum PREPS-Infection Stress, Table S20. Preliminary GLM: Demographics and Postpartum PREPS-Positive Appraisal, Table S21. Preliminary GLM: Healthcare Characteristics and Postpartum PREPS-Positive Appraisal, Table S22. Preliminary GLM: Social Support, Coping and Postpartum PREPS-Positive Appraisal.

## Abbreviations

The following abbreviations are used in this manuscript:

GLM	Generalized Linear Model
L&D	Labor and Delivery
OSSS-3	Oslo Social Support Scale
PREPSs	Pandemic-Related Pregnancy Stress Scales
SES	Socioeconomic Status

## References

[1] Hamelin-Brabant L., De Montigny F., Roch G., Deshaies M.-H., Mbourou-Azizah G., Borgès Da Silva R., Comeau Y., Fournier C. (2015). Vulnérabilité périnatale et soutien social en période postnatale: Une revue de la littérature. Santé Publique.

[2] Chalmers B., Dzakpasu S., Heaman M., Kaczorowski J. (2008). The Canadian Maternity Experiences Survey: An Overview of Findings. J. Obstet. Gynaecol. Can..

[3] Mori E., Tsuchiya M., Maehara K., Iwata H., Sakajo A., Tamakoshi K. (2017). Fatigue, depression, maternal confidence, and maternal satisfaction during the first month postpartum: A comparison of Japanese mothers by age and parity. Int. J. Nurs. Pract..

[4] Bai J., Wong F.W.S., Bauman A., Mohsin M. (2002). Parity and pregnancy outcomes. Am. J. Obstet. Gynecol..

[5] Tomfohr-Madsen L., Cameron E.E., Dunkel Schetter C., Campbell T., O’Beirne M., Letourneau N., Giesbrecht G.F. (2019). Pregnancy anxiety and preterm birth: The moderating role of sleep. Health Psychol..

[6] Preis H., Mahaffey B., Heiselman C., Lobel M. (2020). Vulnerability and resilience to pandemic-related stress among U.S. women pregnant at the start of the COVID-19 pandemic. Soc. Sci. Med..

[7] Preis H., Mahaffey B., Heiselman C., Lobel M. (2020). Pandemic-related pregnancy stress and anxiety among women pregnant during the coronavirus disease 2019 pandemic. Am. J. Obstet. Gynecol. MFM.

[8] Chalmers B., Aziz K., Biringer A., Ciofani L., Di Lallo S., LeDrew M., Menard L.M., Miller K.J., Nemrava J. (2017). Family-centered maternity and newborn care in Canada: Underlying philosophy and principles. Family-Centred Maternity and Newborn Care: National Guidelines.

[9] Brady S., Lee N., Gibbons K., Bogossian F. (2019). Woman-centred care: An integrative review of the empirical literature. Int. J. Nurs. Stud..

[10] Fontein-Kuipers Y., De Groot R., Van Staa A. (2018). Woman-centered care 2.0: Bringing the concept into focus. Eur. J. Midwifery.

[11] Rice K.F., Williams S.A. (2022). Making good care essential: The impact of increased obstetric interventions and decreased services during the COVID-19 pandemic. Women Birth.

[12] Rice K., Williams S. (2021). Women’s postpartum experiences in Canada during the COVID-19 pandemic: A qualitative study. Can. Med. Assoc. Open Access J..

[13] Khoury J.E., Atkinson L., Bennett T., Jack S.M., Gonzalez A. (2022). Prenatal distress, access to services, and birth outcomes during the COVID-19 pandemic: Findings from a longitudinal study. Early Hum. Dev..

[14] Rudrum S. (2021). Pregnancy During the Global COVID-19 Pandemic: Canadian Experiences of Care. Front. Sociol..

[15] Groulx T., Bagshawe M., Giesbrecht G., Tomfohr-Madsen L., Hetherington E., Lebel C.A. (2021). Prenatal Care Disruptions and Associations with Maternal Mental Health During the COVID-19 Pandemic. Front. Glob. Women’s Health.

[16] Vasilevski V., Sweet L., Bradfield Z., Wilson A.N., Hauck Y., Kuliukas L., Homer C.S.E., Szabo R.A., Wynter K. (2022). Receiving maternity care during the COVID-19 pandemic: Experiences of women’s partners and support persons. Women Birth.

[17] Detsky A.S., Bogoch I.I. (2022). COVID-19 in Canada—The Fourth Through Seventh Waves. JAMA Health Forum.

[18] Detsky A.S., Bogoch I.I. (2020). COVID-19 in Canada: Experience and Response. JAMA.

[19] Detsky A.S., Bogoch I.I. (2021). COVID-19 in Canada: Experience and Response to Waves 2 and 3. JAMA.

[20] Canadian Institute for Health Information Canadian COVID-19 Intervention Timeline. https://www.cihi.ca/en/canadian-covid-19-intervention-timeline.

[21] Dol J., Hughes B., Aston M., McMillan D., Tomblin Murphy G., Campbell-Yeo M. (2023). Impact of COVID -19 restrictions on the postpartum experience of women living in Eastern Canada during the early pandemic period: A cross-sectional study. J Nurs. Scholarsh..

[22] Sullivan E., Cameron A., Kornelsen J. (2022). Rural Residents’ Perinatal Experiences During the Initial Months of the COVID-19 Pandemic: A Qualitative Study in British Columbia. J. Midwife Women’s Health.

[23] Racine N., Hetherington E., McArthur B.A., McDonald S., Edwards S., Tough S., Madigan S. (2021). Maternal depressive and anxiety symptoms before and during the COVID-19 pandemic in Canada: A longitudinal analysis. Lancet Psychiatry.

[24] Kolker S., Biringer A., Bytautas J., Blumenfeld H., Kukan S., Carroll J.C. (2021). Pregnant during the COVID-19 pandemic: An exploration of patients’ lived experiences. BMC Pregnancy Childbirth.

[25] Rice K., Williams S. (2024). Partner Exclusion from Childbirth During COVID-19 in Canada: Implications for Theory and Policy. Med. Anthropol..

[26] Reynolds K.A., Pankratz L., Cameron E.E., Roos L.E., Giesbrecht G.F., Lebel C., Tomfohr-Madsen L.M. (2022). Pregnancy during the COVID-19 pandemic: A qualitative examination of ways of coping. Arch. Women’s Ment. Health.

[27] Preis H., Mahaffey B., Lobel M. (2020). Psychometric properties of the Pandemic-Related Pregnancy Stress Scale (PREPS). J. Psychosom. Obstet. Gynecol..

[28] Levinson A., Mahaffey B., Lobel M., Preis H. (2022). Development and psychometric properties of the Pandemic-Related Postpartum Stress Scale (PREPS-PP). J. Psychosom. Obstet. Gynecol..

[29] Garcia-Silva J., Caracuel A., Lozano-Ruiz A., Alderdice F., Lobel M., Perra O., Caparros-Gonzalez R.A. (2021). Pandemic-related pregnancy stress among pregnant women during the COVID-19 pandemic in Spain. Midwifery.

[30] Ilska M., Kołodziej-Zaleska A., Brandt-Salmeri A., Preis H., Lobel M. (2021). Pandemic Stress and Its Correlates among Pregnant Women during the Second Wave of COVID-19 in Poland. Int. J. Environ. Res. Public Health.

[31] Penengo C., Colli C., Cesco M., Croccia V., Degano M., Ferreghini A., Garzitto M., Lobel M., Preis H., Sala A. (2022). Stress, Coping, and Psychiatric Symptoms in Pregnant Women in Outpatient Care During the 2021 Second-Wave COVID-19 Pandemic. Front. Psychiatry.

[32] Riquelme-Gallego B., Ordoñez-Carrasco J.L., Suárez-Yera C., Rojas-Tejada A.J., Preis H., Lobel M., Mahaffey B., Castro R.A., Atzil S., Balestrieri M. (2025). Assessment of pandemic-related pregnancy stress from seven western countries using Rasch analyses. J. Psychiatr. Res..

[33] Siafaka V., Tsonis O., Christogiannis C., Kontouli K.-M., Margariti K., Barbalia Z., Flindris S., Manifava E., Paschopoulou K.I., Tzioras S. (2023). Psychometric properties of the Greek versions of the Pandemic-Related Pregnancy Stress Scale and the Pandemic-Related Postpartum Stress Scale and associated risk factors during the second year of the COVID-19 pandemic. BJPsych Open.

[34] Schaal N.K., Marca-Ghaemmaghami P.L., Preis H., Mahaffey B., Lobel M., Amiel Castro R. (2021). The German version of the pandemic-related pregnancy stress scale: A validation study. Eur. J. Obstet. Gynecol. Reprod. Biol..

[35] Yirmiya K., Yakirevich-Amir N., Preis H., Lotan A., Atzil S., Reuveni I. (2021). Women’s Depressive Symptoms during the COVID-19 Pandemic: The Role of Pregnancy. Int. J. Environ. Res. Public Health.

[36] Riquelme-Gallego B., Martínez-Vázquez S., Caparros-Gonzalez R.A. (2025). Pandemic-related stress in pregnant women during the first COVID-19 lockdown and neonatal development. J. Reprod. Infant Psychol..

[37] Ilska M., Kołodziej-Zaleska A., Brandt-Salmeri A., Preis H., Lobel M. (2025). Changes in Fear of Childbirth and Its Predictors Over Three COVID-19 Pandemic Waves in Poland. Birth.

[38] Colli C., Penengo C., Garzitto M., Driul L., Sala A., Degano M., Preis H., Lobel M., Balestrieri M. (2021). Prenatal Stress and Psychiatric Symptoms During Early Phases of the COVID-19 Pandemic in Italy. Int. J. Women’s Health.

[39] Penengo C., Colli C., Garzitto M., Driul L., Sala A., Degano M., Preis H., Lobel M., Balestrieri M. (2021). Psychometric properties of the Italian version of the Pandemic-Related Pregnancy Stress Scale (PREPS) and its correlation with anxiety and depression. J. Affect. Disord..

[40] Ilska M., Brandt-Salmeri A., Kołodziej-Zaleska A., Preis H., Rehbein E., Lobel M. (2022). Anxiety among pregnant women during the first wave of the COVID-19 pandemic in Poland. Sci. Rep..

[41] Government of Canada The Canadian Maternity Experiences Survey: Executive Summary. https://www.canada.ca/en/public-health/services/injury-prevention/health-surveillance-epidemiology-division/maternal-infant-health/canadian-maternity-experiences-survey.html.

[42] Kocalevent R.-D., Berg L., Beutel M.E., Hinz A., Zenger M., Härter M., Nater U., Brähler E. (2018). Social support in the general population: Standardization of the Oslo social support scale (OSSS-3). BMC Psychol..

[43] Carver C.S. (1997). You want to measure coping but your protocol’ too long: Consider the brief cope. Int. J. Behav. Med..

[44] NovoPsych Coping Orientation to Problems Experienced Inventory (Brief COPE). https://novopsych.com/assessments/formulation/brief-cope/.

[45] Science of Behavior Change. Measures. Brief COPE. https://repository.scienceofbehaviorchange.org.

[46] Hegarty D., Buchanan B. (2021). The Value of NovoPsych Data—New Norms for the Brief-COPE. https://novopsych.com/news/the-value-of-novopsych-data-new-norms-for-the-brief-cope/.

[47] Ollivier R., Aston M., Price S., Sim M., Benoit B., Joy P., Iduye D., Nassaji N.A. (2021). Mental Health & Parental Concerns during COVID-19: The Experiences of New Mothers Amidst Social Isolation. Midwifery.

[48] Preis H., Mahaffey B., Lobel M. (2021). The role of pandemic-related pregnancy stress in preference for community birth during the beginning of the COVID-19 pandemic in the United States. Birth.

[49] Lobel M., Preis H., Mahaffey B., Schaal N.K., Yirmiya K., Atzil S., Reuveni I., Balestrieri M., Penengo C., Colli C. (2022). Common model of stress, anxiety, and depressive symptoms in pregnant women from seven high-income Western countries at the COVID-19 pandemic onset. Soc. Sci. Med..

[50] Doucet A., Mathieu S., McKay L. (2020). Reconceptualizing Parental Leave Benefits in COVID-19 Canada: From Employment Policy to Care and Social Protection Policy. Can. Public Policy.

[51] Dahlen H.G., Barclay L.M., Homer C. (2008). Preparing for the First Birth: Mothers’ Experiences at Home and in Hospital in Australia. J. Perinat. Educ..

[52] McCarthy M., Houghton C., Matvienko-Sikar K. (2021). Women’s experiences and perceptions of anxiety and stress during the perinatal period: A systematic review and qualitative evidence synthesis. BMC Pregnancy Childbirth.

[53] Chen T., Laplante D.P., Elgbeili G., Brunet A., Simcock G., Kildea S., King S. (2020). Coping During Pregnancy Following Exposure to a Natural Disaster: The QF2011 Queensland Flood Study. J. Affect. Disord..

[54] Heaman M.I., Moffatt M., Elliott L., Sword W., Helewa M.E., Morris H., Gregory P., Tjaden L., Cook C. (2014). Barriers, motivators and facilitators related to prenatal care utilization among inner-city women in Winnipeg, Canada: A case–control study. BMC Pregnancy Childbirth.

[55] Redshaw M., Heikkilä K. (2010). Delivered with Care. A National Survey of Women’s Experience of Maternity Care 2010.

[56] O’Sullivan T.L., Amaratunga C., Phillips K.P., Corneil W., O’Connor E., Lemyre L., Dow D. (2009). If Schools Are Closed, Who Will Watch Our Kids? Family Caregiving and Other Sources of Role Conflict among Nurses during Large-Scale Outbreaks. Prehosp. Disaster. Med..

[57] O’Sullivan T.L., Phillips K.P. (2019). From SARS to pandemic influenza: The framing of high-risk populations. Nat. Hazards.

[58] Ilska M., Kołodziej-Zaleska A., Brandt-Salmeri A., Preis H., Lobel M. (2021). Pandemic-related pregnancy stress assessment–Psychometric properties of the Polish PREPS and its relationship with childbirth fear. Midwifery.

[59] Shaw-Churchill S., Phillips K.P. (2025). Pandemic Pregnancy Experiences and Risk Mitigation Behaviors: COVID-19 Vaccination Uptake in Canada. Int. J. Environ. Res. Public Health.

[60] Statistics Canada (2016). Population and Dwelling Count Highlight Tables, 2016 Census. https://www12.statcan.gc.ca/census-recensement/2016/dp-pd/hlt-fst/pd-pl/Table.cfm?Lang=Eng&T=101&SR=1&S=10&O=D#tPopDwell.

[61] Barry K., Fernández-García S., Khashaba A., Ruiz-Calvo G., Roncal Redin M., Mahmoud G., Yap M., King Y., Zhou D., Mamey M. (2025). Global maternal mortality associated with SARS-CoV-2 infection: A systematic review and meta-analysis. BMJ Glob. Health.

[62] Simbar M., Nazarpour S., Sheidaei A. (2023). Evaluation of pregnancy outcomes in mothers with COVID-19 infection: A systematic review and meta-analysis. J. Obstet. Gynaecol..

[63] John J.N., Gorman S., Scales D., Gorman J. (2025). Online Misleading Information About Women’s Reproductive Health: A Narrative Review. J. Gen. Intern. Med..

[64] Durowaye T.D., Rice A.R., Konkle A.T.M., Phillips K.P. (2022). Public health perinatal promotion during COVID-19 pandemic: A social media analysis. BMC Public Health.

[65] Downe S., Finlayson K., Oladapo O., Bonet M., Gülmezoglu A.M. (2018). What matters to women during childbirth: A systematic qualitative review. PLoS ONE.

[66] Pan-Canadian Public Health Network Council (2018). Canadian Pandemic Influenza Preparedness: Planning Guidance for the Health Sector. Public Health Agency of Canada. https://www.canada.ca/en/public-health/services/flu-influenza/canadian-pandemic-influenza-preparedness-planning-guidance-health-sector.html.

[67] National Advisory Committee on Immunization (NACI) (2020). Preliminary Guidance on Key Populations for Early COVID-19 Immunization. https://www.canada.ca/en/public-health/services/immunization/national-advisory-committee-on-immunization-naci/guidance-key-populations-early-covid-19-immunization.html.

[68] Truth and Reconciliation Commission of Canada (2015). Truth and Reconciliation Commission of Canada: Calls to Action. National Centre for Truth and Reconciliation. https://nctr.ca/about/history-of-the-trc/truth-and-reconciliation-commission-of-canada/.

[69] Bohren M.A., Berger B.O., Munthe-Kaas H., Tunçalp Ö. (2019). Perceptions and experiences of labour companionship: A qualitative evidence synthesis. Cochrane Database Syst. Rev..

[70] Dol J., Richardson B., Grant A., Aston M., McMillan D., Tomblin Murphy G., Campbell-Yeo M. (2021). Influence of parity and infant age on maternal self-efficacy, social support, postpartum anxiety, and postpartum depression in the first six months in the Maritime Provinces, Canada. Birth.

[71] Tani F., Castagna V. (2017). Maternal social support, quality of birth experience, and post-partum depression in primiparous women. J. Matern.-Fetal Neonatal Med..

[72] Bedaso A., Adams J., Peng W., Sibbritt D. (2021). The relationship between social support and mental health problems during pregnancy: A systematic review and meta-analysis. Reprod Health.

[73] Lancaster C.A., Gold K.J., Flynn H.A., Yoo H., Marcus S.M., Davis M.M. (2010). Risk factors for depressive symptoms during pregnancy: A systematic review. Am. J. Obstet. Gynecol..

[74] Bérard A., Gorgui J., Tchuente V., Lacasse A., Gomez Y.-H., Côté S., King S., Muanda F., Mufike Y., Boucoiran I. (2022). The COVID-19 Pandemic Impacted Maternal Mental Health Differently Depending on Pregnancy Status and Trimester of Gestation. Int. J. Environ. Res. Public Health.

[75] Khoury J.E., Atkinson L., Bennett T., Jack S.M., Gonzalez A. (2021). Coping strategies mediate the associations between COVID-19 experiences and mental health outcomes in pregnancy. Arch. Women’s Ment. Health.

[76] Guardino C.M., Dunkel Schetter C. (2014). Coping during pregnancy: A systematic review and recommendations. Health Psychol. Rev..

[77] Yali A.M., Lobel M. (1999). Coping and distress in pregnancy: An investigation of medically high risk women. J. Psychosom. Obstet. Gynecol..

