# Supplementary material for: Predictors of COVID-19 Pandemic-Related Pregnancy Stress: Prenatal and Postpartum Experiences in Canada
Source: Int J Environ Res Public Health. 2025 Aug 20;22(8):1302. doi: 10.3390/ijerph22081302 (PMC12386160; doi:10.3390/ijerph22081302)
Supplement: Supplementary file 1 [file ijerph-22-01302-s001.zip › ijerph-3676134-supplementary.pdf]

Predictors of COVID-19 pandemic-related pregnancy stress: Prenatal and postpartum experiences in Canada

Table S1. Mean Prenatal Psychometric Scales Scores by Respondent Characteristics

Characteristic	OSSS-3	Problem-Focused Coping	Emotion-Focused Coping	Avoidant Coping
Indigenous	8.4 ± 4.08	2.93 ± 0.41	2.43 ± 0.47	1.71 ± 0.59
Racialized	7.5 ± 2.83	2.76 ± 0.62	2.33 ± 0.50	1.71 ± 0.50
Immigrant	8.0 ± 2.25	2.58 ± 0.51	2.43 ± 0.51	1.65 ± 0.22
**Parity**				
Primigravid	8.45 ± 2.72	2.57 ± 0.54	2.33 ± 0.42	1.71 ± 0.38
Multigravid	7.86 ± 2.93	2.63 ± 0.59	2.35 ± 0.43	1.71 ± 0.44
**Education**				
Some Highschool/Highschool	7.66 ± 3.32	2.45 ± 0.61	2.36 ± 0.42	1.78 ± 0.52
College	7.91 ± 2.66	2.53 ± 0.53	2.32 ± 0.44	1.75 ± 0.44
University	8.11 ± 2.81	2.59 ± 0.57	2.33 ± 0.40	1.70 ± 0.38
Post-Graduate Studies	8.93 ± 2.72	2.73 ± 0.54	2.36 ± 0.45	1.66 ± 0.36
**Income**				
<$50,000	6.63 ± 3.22	2.67 ± 0.53	2.46 ± 0.45	1.91 ± 0.66
$50,000-79,999	7.25 ± 2.88	2.47 ± 0.61	2.45 ± 0.40	1.79 ± 0.52
$80,000-$99,999	7.78 ± 2.75	2.48 ± 0.51	2.24 ± 0.46	1.72 ± 0.41
$100,000-149,999	8.26 ± 2.60	2.61 ± 0.58	2.31 ± 0.42	1.65 ± 0.29
>$1500,000	8.90 ± 2.89	2.66 ± 0.57	2.35 ± 0.40	1.71 ± 0.40

SD- standard deviation, OSSS-3 Oslo Social Support Scale

Table S2. Mean Prenatal PREPS Scores by Respondent Characteristics

Characteristic	Preparedness Stress	Infection Stress	Positive Appraisal
Indigenous	3.2 ± 1.1	3.4 ± 0.89	2.68 ± 0.97
Racialized	3.06 ± 1.04	3.39 ± 0.99	2.15 ± 0.82
Immigrant	3.11 ± 1.06	3.17 ± 1.16	2.12 ± 0.83
**Parity**			
Primigravid	2.92 ± 1.00	3.02 ± 0.97	2.14 ± 0.84
Multigravid	3.02 ± 1.01	3.03 ± 1.13	2.03 ± 0.88
**Education**			
Some Highschool/Highschool	2.94 ± 0.98	2.63 ± 1.16	2.04 ± 0.73
College	3.04 ± 1.07	3.05 ± 1.07	2.22 ± 0.84
University	2.97 ± 0.98	3.12 ± 0.97	2.05 ± 0.86
Post-Graduate Studies	2.86 ± 0.98	3.00 ± 1.04	2.07 ± 0.90
**Income**			
<$50,000	3.28 ± 0.79	2.94 ± 1.16	2.36 ± 1.03
$50,000-$79,999	3.29 ± 1.10	3.01 ± 1.22	2.07 ± 0.88
$80,000-$99,999	2.98 ± 1.03	2.88 ± 1.02	2.26 ± 0.85
$100,000-$149,999	2.90 ± 0.94	3.06 ± 0.97	2.09 ± 0.88
>$150,000	2.80 ± 1.00	3.10 ± 1.02	1.99 ± 0.82

SD- standard deviation

Table S3. Preliminary GLM: Demographics and Prenatal PREPS-Preparedness Stress

Variables	B	Lower CI	Upper CI	p-value
Indigenous (not Indigenous)	0.45	-0.06	0.95	0.08
Racialized (not racialized)	0.12	-0.34	0.58	0.60
Immigrant (Canadian)	-0.03	-0.54	0.47	0.90
Education -Some Highschool/Highschool (post-graduate)	-0.30	-0.73	0.12	0.16
Education- College (post-graduate)	-0.03	-0.33	0.27	0.85
Education- University (post-graduate)	0.04	-0.22	0.30	0.76
Income <$50,000 (>$150,000)*	0.55	0.05	1.05	0.03
Income $50,000 - $79,999 (>$150,000)*	0.47	0.09	0.85	0.02
Income $80,000 - $99,999 (>$150,000)	0.09	-0.23	0.40	0.58
Income $100,000 - $149,999 (>$150,000)	0.08	-0.17	0.32	0.55
Age*	-0.03	-0.06	-0.01	0.02

Goodness of Fit: deviance/df=0.96 (df=350); Likelihood ratio $\chi^2(11)=22.3$; p=0.02. *p<0.05. Reference categories shown in brackets. CI- 95% Confidence Interval

Table S4. Preliminary GLM: Healthcare Characteristics and Prenatal PREPS-Preparedness Stress

Variables	B	Lower CI	Upper CI	p-value
Primigravid (multigravid)	0.00	-0.21	0.21	0.98
HCP Ob/Gyn (midwife)	-0.13	-0.39	0.14	0.35
HCP Family Physician (midwife)	-0.15	-0.45	0.16	0.35
Partner Attended Prenatal Appointments (could not attend)	-0.09	-0.33	0.16	0.48
Prenatal Appointments Cancelled (not cancelled)***	0.54	0.31	0.77	<0.001
Prenatal Appointments Rescheduled as Virtual/Phone (not rescheduled)	0.02	-0.19	0.23	0.82
Satisfied Prenatal Care Provider (not satisfied)***	-0.53	-0.78	-0.28	<0.001
Prenatal Care Province-West (Atlantic)	-0.03	-0.40	0.35	0.90
Prenatal Care Province-Prairies (Atlantic)	0.24	-0.10	0.57	0.17
Prenatal Care Province-Ontario (Atlantic)	0.10	-0.18	0.38	0.47
Prenatal Care Province-Quebec (Atlantic)	-0.06	-0.49	0.37	0.78
Location Prenatal Care -Hospital (birthing/community centre/home)	0.25	-0.17	0.67	0.24
Location Prenatal Care -Clinic/Primary Care (birthing/community centre/home)	0.13	-0.23	0.49	0.48
COVID Hot-Spot (low-no COVID)*	0.36	0.08	0.65	0.01
COVID Moderate (low-no COVID)	0.18	-0.09	0.44	0.20
Prenatal Education (no)	-0.04	-0.26	0.18	0.71

Goodness of Fit: deviance/df=0.87 (df=347); Likelihood ratio $\chi^2(16)=67.1$; p<0.001. *p<0.05; ***p<0.001. Reference categories shown in brackets. CI- 95% Confidence Interval

Table S5. Preliminary GLM: Social Support, Coping and Prenatal PREPS-Preparedness Stress

Variables	B	Lower CI	Upper CI	p-value
OSS-3**	-0.054	-0.090	-0.018	0.003
COPE-Problem-Based Coping Subscale	0.092	-0.118	0.302	0.391
COPE-Emotion-Based Coping Subscale	0.043	-0.251	0.337	0.773
COPE- Avoidant Based Coping Subscale***	0.859	0.587	1.130	<0.001

Goodness of Fit: deviance/df=0.87 (df=338); Likelihood ratio $\chi^2(4)=60.0$; p<0.001. **p<0.01; ***p<0.001. Reference categories shown in brackets. CI- 95% Confidence Interval

Table S6. Preliminary GLM: Demographics and Prenatal PREPS-Infection Stress

Variables	B	Lower CI	Upper CI	p-value
Indigenous (not Indigenous)*	0.594	0.070	1.118	0.026
Racialized (not racialized)	0.472	-0.007	0.951	0.053
Immigrant (Canadian)	-0.232	-0.758	0.294	0.387
Education -Some Highschool/Highschool (post-graduate)	-0.428	-0.873	0.018	0.060
Education- College (post-graduate)	0.188	-0.125	0.501	0.240
Education- University (post-graduate)	0.174	-0.095	0.443	0.205
Income <$50,000 (>$150,000)	0.017	-0.492	0.526	0.947
Income $50,000 - $79,999 (>$150,000)	-0.003	-0.398	0.391	0.987
Income $80,000 - $99,999 (>$150,000)	-0.279	-0.605	0.046	0.093
Income $100,000 - $149,999 (>$150,000)	-0.006	-0.262	0.251	0.965
Age	0.019	-0.008	0.047	0.162

Goodness of Fit: deviance/df=1.03 (df=351); Likelihood ratio $\chi^2(11)=23.05$; p=0.017. *p<0.05. Reference categories shown in brackets. CI- 95% Confidence Interval

Table S7. Preliminary GLM: Healthcare Characteristics and Prenatal PREPS -Infection Stress

Variables	B	Lower CI	Upper CI	p-value
Primigravid (multigravid)	0.042	-0.181	0.266	0.710
HCP Ob/Gyn (midwife)	-0.027	-0.310	0.256	0.853
HCP Family Physician (midwife)	-0.231	-0.559	0.097	0.168
Partner Attended Prenatal Appointments (could not attend)*	-0.288	-0.549	-0.028	0.030
Prenatal Appointments Cancelled (not cancelled)*	0.272	0.024	0.520	0.031
Prenatal Appointments Rescheduled as Virtual/Phone(not rescheduled)	-0.027	-0.251	0.198	0.816
Satisfied Prenatal Care Provider (not satisfied)	0.135	-0.135	0.404	0.327
Prenatal Care Province-West (Atlantic)	-0.146	-0.551	0.258	0.479
Prenatal Care Province-Prairies (Atlantic)*	-0.401	-0.761	-0.041	0.029
Prenatal Care Province-Ontario (Atlantic)	-0.232	-0.530	0.067	0.128
Prenatal Care Province-Quebec (Atlantic)	-0.077	-0.542	0.387	0.744
Location Prenatal Care -Hospital (birthing/community centre/home)	0.082	-0.368	0.532	0.721
Location Prenatal Care -Clinic/Primary Care (birthing/community centre/home)	0.070	-0.322	0.462	0.727
COVID Hot-Spot (low-no COVID)***	0.643	0.337	0.950	p<0.001
COVID Moderate (low-no COVID)**	0.416	0.129	0.704	0.004
Prenatal Education (no)	-0.051	-0.284	0.182	0.668

Goodness of Fit: deviance/df=1.01 (df=348); Likelihood ratio $\chi^2(16)=39.54$; p=0.001. *p<0.05; **p<0.01; ***p<0.001. Reference categories shown in brackets. CI- 95% Confidence Interval

Table S8. Preliminary GLM: Social Support, Coping and Prenatal PREPS-Infection Stress

Variables	B	Lower CI	Upper CI	p-value
OSS-3	-0.024	-0.063	0.015	0.225
COPE-Problem-Based Coping Subscale	0.184	-0.043	0.411	0.112
COPE-Emotion-Based Coping Subscale	-0.073	-0.392	0.245	0.652
COPE- Avoidant Based Coping Subscale**	0.447	0.154	0.740	0.003

Goodness of Fit: deviance/df=1.02 (df=339); Likelihood ratio $\chi^2(4)=14.4$; p=0.006. ** p<0.01. Reference categories shown in brackets. CI- 95% Confidence Interval

Table S9. Preliminary GLM: Demographics and Prenatal PREPS-Positive Appraisal

Variables	B	Lower CI	Upper CI	p-value
Indigenous (not Indigenous)**	0.746	0.306	1.187	0.001
Racialized (not racialized)	0.144	-0.259	0.546	0.485
Immigrant (Canadian)	-0.140	-0.582	0.302	0.534
Education -Some Highschool/Highschool (post-graduate)	-0.083	-0.457	0.291	0.664
Education- College (post-graduate)	0.145	-0.118	0.408	0.280
Education- University (post-graduate)	-0.008	-0.234	0.219	0.947
Income <$50,000 (>$150,000)	0.314	-0.114	0.742	0.150
Income $50,000 - $79,999 (>$150,000)	-0.083	-0.415	0.248	0.622
Income $80,000 - $99,999 (>$150,000)	0.204	-0.070	0.477	0.145
Income $100,000 - $149,999 (>$150,000)	0.069	-0.146	0.285	0.529
Age	-0.009	-0.032	0.014	0.422

Goodness of Fit: deviance/df=0.73 (df=351); Likelihood ratio $\chi^2(11)=20.85$; p=0.035. **p<0.01. Reference categories shown in brackets. CI- 95% Confidence Interval

Table S10. Preliminary GLM: Healthcare Characteristics and Prenatal PREPS -Positive Appraisal

Variables	B	Lower CI	Upper CI	p-value
Primigravid (multigravid)	0.06	-0.13	0.25	0.53
HCP Ob/Gyn (midwife)	-0.07	-0.31	0.18	0.60
HCP Family Physician (midwife)	-0.05	-0.33	0.23	0.72
Partner Attended Prenatal Appointments (could not attend)	0.09	-0.14	0.31	0.44
Prenatal Appointments Cancelled (not cancelled)	-0.12	-0.33	0.10	0.29
Prenatal Appointments Rescheduled as Virtual/Phone (not rescheduled)	-0.11	-0.30	0.09	0.28
Satisfied Prenatal Care Provider (not satisfied)	0.07	-0.16	0.30	0.56
Prenatal Care Province-West (Atlantic)	-0.18	-0.53	0.16	0.30
Prenatal Care Province-Prairies (Atlantic)	0.04	-0.27	0.34	0.82
Prenatal Care Province-Ontario (Atlantic)	0.04	-0.22	0.29	0.78
Prenatal Care Province-Quebec (Atlantic)	-0.18	-0.58	0.22	0.37
Location Prenatal Care -Hospital (birthing/community centre/home)	-0.11	-0.50	0.28	0.57
Location Prenatal Care -Clinic/Primary Care (birthing/community centre/home)	-0.07	-0.40	0.27	0.70
COVID Hot-Spot (low-no COVID)*	0.30	0.03	0.56	0.03
COVID Moderate (low-no COVID)	0.12	-0.13	0.37	0.34
Prenatal Education (no)	0.04	-0.16	0.24	0.71

Goodness of Fit: deviance/df=0.74 (df=348); Likelihood ratio $\chi^2(16)=13.60$; p=0.63 - variables shown did not meet threshold for inclusion in final model. *p<0.05. Reference categories shown in brackets. CI- 95% Confidence Interval

Table S11. Preliminary GLM: Social Support, Coping and Prenatal PREPS-Positive Appraisal

Variables	B	Lower CI	Upper CI	p-value
OSS-3	0.005	-0.027	0.037	0.768
COPE-Problem-Based Coping Subscale**	0.328	0.141	0.515	0.001
COPE-Emotion-Based Coping Subscale	0.177	-0.086	0.440	0.186
COPE- Avoidant Based Coping Subscale	-0.017	-0.259	0.225	0.888

Goodness of Fit: deviance/df=0.70 (df=339); Likelihood ratio $\chi^2(4)=26.1$; p<0.001. ** p<0.01. Reference categories shown in brackets. CI- 95% Confidence Interval

Table S12. Mean Postpartum Psychometric Scales Scores by Respondent Characteristics

Characteristic	OSSS-3	Problem-Focused Coping	Emotion-Focused Coping	Avoidant Coping
Indigenous	8.58 ± 3.68	2.86 ± 0.30	2.44 ± 0.51	1.69 ± 0.49
Racialized	7.14 ± 3.01	2.86 ± 0.64	2.32 ± 0.51	1.65 ± 0.38
Immigrant	7.60 ± 2.22	2.48 ± 0.65	2.38 ± 0.54	1.59 ± 0.25
**Parity**				
Primigravid	8.43 ± 2.76	2.61 ± 0.58	2.32 ± 0.41	1.66 ± 0.36
Multigravid	8.29 ± 2.86	2.70 ± 0.56	2.36 ± 0.43	1.65 ± 0.36
**Education**				
Some Highschool/Highschool	7.62 ± 3.33	2.63 ± 0.51	2.46 ± 0.49	1.72 ± 0.57
College	7.95 ± 2.64	2.58 ± 0.59	2.33 ± 0.39	1.67 ± 0.32
University	8.32 ± 2.77	2.61 ± 0.56	2.31 ± 0.39	1.68 ± 0.36
Post-Graduate Studies	9.11 ± 2.79	2.79 ± 0.58	2.38 ± 0.46	1.60 ± 0.32
**Income**				
<$50,000	7.55 ± 3.56	2.72 ± 0.61	2.48 ± 0.47	1.82 ± 0.61
$50,000-79,999	6.67 ± 3.24	2.46 ± 0.45	2.50 ± 0.34	1.68 ± 0.43
$80,000-$99,999	7.91 ± 2.83	2.47 ± 0.44	2.26 ± 0.39	1.61 ± 0.29
$100,000-149,999	8.66 ± 2.31	2.69 ± 0.61	2.32 ± 0.44	1.63 ± 0.27
>$150,000	8.89 ± 2.94	2.71 ± 0.63	2.33 ± 0.41	1.68 ± 0.40

SD- standard deviation, OSSS-3 Oslo Social Support Scale

Table S13. Mean Postpartum PREPS Scores by Respondent Characteristics

Characteristic	Preparedness Stress	Infection Stress	Positive Appraisal
Indigenous	3.18 ± 0.91	3.43 ± 0.79	3.30 ± 1.17
Racialized	3.05 ± 0.84	3.02 ± 0.82	2.58 ± 1.17
Immigrant	2.59 ± 1.09	2.39 ± 0.98	2.37 ± 1.09
**Parity**			
Primigravid	3.02 ± 1.02	2.98 ± 1.06	2.60 ± 1.02
Multigravid	3.31 ± 0.91	3.10 ± 1.09	2.55 ± 1.13
**Education**			
Some Highschool/Highschool	2.78 ± 0.64	2.20 ± 0.86	2.61 ± 1.02
College	3.21 ± 1.02	3.21 ± 1.20	2.67 ± 1.00
University	3.19 ± 0.96	3.11 ± 0.97	2.60 ± 1.10
Post-Graduate Studies	3.08 ± 1.06	2.89 ± 1.06	2.42 ± 1.12
**Income**			
<$50,000	2.94 ± 0.77	2.70 ± 1.16	2.88 ± 0.90
$50,000-$79,999	3.27 ± 0.99	2.76 ± 1.06	2.98 ± 1.02
$80,000-$99,999	3.15 ± 1.00	3.13 ± 1.09	2.73 ± 1.17
$100,000-$149,999	3.26 ± 1.05	3.06 ± 1.15	2.51 ± 1.12
>$150,000	3.06 ± 0.93	3.11 ± 0.98	2.50 ± 1.02

SD- standard deviation

Table S14. Preliminary GLM: Demographics and Postpartum PREPS-Preparedness Stress

Variables	B	Lower CI	Upper CI	p-value
Indigenous (not Indigenous)	0.22	-0.46	0.90	0.52
Racialized (not racialized)	0.32	-0.32	0.97	0.33
Immigrant (Canadian)*	-0.89	-1.63	-0.14	0.02
Education -Some Highschool/Highschool (post-graduate)	-0.42	-1.12	0.28	0.24
Education- College (post-graduate)	-0.04	-0.46	0.37	0.84
Education- University (post-graduate)	0.09	-0.28	0.45	0.64
Income <$50,000 (>$150,000)	0.09	-0.59	0.78	0.79
Income $50,000 - $79,999 (>$150,000)	0.46	-0.11	1.03	0.12
Income $80,000 - $99,999 (>$150,000)	0.19	-0.28	0.66	0.42
Income $100,000 - $149,999 (>$150,000)	0.26	-0.09	0.60	0.15
Age	0.02	-0.02	0.06	0.39

Goodness of Fit: deviance/df=0.94 (df=166); Likelihood ratio $\chi^2(11)=10.89$; p=0.45 – variables shown did not meet threshold for inclusion in final model. *p<0.05. Reference categories shown in brackets. CI- 95% Confidence Interval

Table S15. Preliminary GLM: Healthcare Characteristics and Postpartum PREPS -Preparedness Stress

Variables	B	Lower CI	Upper CI	p-value
Primigravid (multigravid)*	-0.38	-0.66	-0.10	0.01
Vaginal Delivery (C-section)	-0.02	-0.32	0.29	0.91
HCP Ob/Gyn (midwife)	0.38	-0.07	0.83	0.10
HCP Family Physician (midwife)	0.02	-0.64	0.67	0.96
Birthplace of Choice (no choice)	-0.38	-0.85	0.09	0.11
Satisfied Prenatal Care Provider (not satisfied)	-0.28	-0.81	0.25	0.31
Support Companion Restricted (not restricted)	0.03	-0.57	0.64	0.92
Prenatal Care Province-West (Atlantic)	-0.32	-0.91	0.26	0.28
Prenatal Care Province-Prairies (Atlantic)	0.10	-0.39	0.59	0.69
Prenatal Care Province-Ontario (Atlantic)	0.20	-0.19	0.59	0.31
Prenatal Care Province-Quebec (Atlantic)	0.13	-0.51	0.78	0.68
COVID Hot-Spot (low-no COVID)	0.32	-0.10	0.74	0.13
COVID Moderate (low-no COVID)	-0.04	-0.45	0.37	0.84

Goodness of Fit: deviance/df=0.91 (df=166); Likelihood ratio $\chi^2(13)=25.62$; p=0.02. *p<0.05. Reference categories shown in brackets. CI- 95% Confidence Interval

Table S16. Preliminary GLM: Social Support, Coping and Postpartum PREPS-Preparedness Stress

Variables	B	Lower CI	Upper CI	p-value
OSS-3	0.00	-0.05	0.05	0.95
COPE-Problem-Based Coping Subscale	0.06	-0.20	0.33	0.65
COPE-Emotion-Based Coping Subscale	-0.22	-0.61	0.17	0.26
COPE- Avoidant Based Coping Subscale***	1.18	0.78	1.57	<0.001

Goodness of Fit: deviance/df=0.82 (df=183); Likelihood ratio $\chi^2(4)=33.66$; p<0.001. ***p<0.001. Reference categories shown in brackets. CI- 95% Confidence Interval

Table S17. Preliminary GLM: Demographics and Postpartum PREPS-Infection Stress

Variables	B	Lower CI	Upper CI	p-value
Indigenous (not Indigenous)	0.44	-0.29	1.17	0.24
Racialized (not racialized)	0.36	-0.34	1.06	0.31
Immigrant (Canadian)*	-1.08	-1.88	-0.27	0.01
Education -Some Highschool/Highschool (post-graduate)	-0.66	-1.42	0.09	0.08
Education- College (post-graduate)	0.35	-0.09	0.80	0.12
Education- University (post-graduate)	0.26	-0.14	0.65	0.20
Income <$50,000 (>$150,000)*	-0.09	-0.82	0.65	0.82
Income $50,000 - $79,999 (>$150,000)*	-0.29	-0.91	0.33	0.35
Income $80,000 - $99,999 (>$150,000)	0.03	-0.47	0.53	0.91
Income $100,000 - $149,999 (>$150,000)	-0.07	-0.44	0.31	0.73
Age*	0.02	-0.02	0.06	0.34

Goodness of Fit: deviance/df=1.10 (df=166); Likelihood ratio $\chi^2(11)=20.24$; p=0.04. *p<0.05. Reference categories shown in brackets. CI- 95% Confidence Interval

Table S18. Preliminary GLM: Healthcare Characteristics and Postpartum PREPS -Infection Stress

Variables	B	Lower CI	Upper CI	p-value
Primigravid (multigravid)	-0.16	-0.46	0.13	0.28
Vaginal Delivery (C-section)	-0.09	-0.41	0.23	0.59
HCP Ob/Gyn (midwife)	0.39	-0.09	0.87	0.11
HCP Family Physician (midwife)	-0.52	-1.21	0.17	0.14
Birthplace of Choice (no choice)	-0.15	-0.64	0.35	0.56
Satisfied Prenatal Care Provider (not satisfied)	0.15	-0.41	0.72	0.60
Support Companion Restricted (not restricted)	0.28	-0.36	0.92	0.39
Prenatal Care Province-West (Atlantic)	-0.47	-1.09	0.15	0.14
Prenatal Care Province-Prairies (Atlantic)***	-0.75	-1.26	-0.23	<0.001
Prenatal Care Province-Ontario (Atlantic)	-0.30	-0.71	0.11	0.16
Prenatal Care Province-Quebec (Atlantic)	0.08	-0.60	0.76	0.81
COVID Hot-Spot (low-no COVID)***	0.73	0.28	1.17	<0.001
COVID Moderate (low-no COVID)	0.36	-0.08	0.79	0.11

Goodness of Fit: deviance/df=1.02 (df=166); Likelihood ratio $\chi^2(13)=33.51$; p=0.001. *p<0.05; p<0.01; ***p<0.001. Reference categories shown in brackets. CI- 95% Confidence Interval

Table S19. Preliminary GLM: Social Support, Coping and Postpartum PREPS-Infection Stress

Variables	B	Lower CI	Upper CI	p-value
OSS-3	0.05	0.00	0.11	0.05
COPE-Problem-Based Coping Subscale	0.06	-0.24	0.37	0.70
COPE-Emotion-Based Coping Subscale	-0.30	-0.75	0.14	0.18
COPE- Avoidant Based Coping Subscale***	0.81	0.36	1.27	<0.001

Goodness of Fit: deviance/df=1.08 (df=183); Likelihood ratio $\chi^2(4)=13.96$; p=0.007. ***p<0.001. Reference categories shown in brackets. CI- 95% Confidence Interval

Table S20. Preliminary GLM: Demographics and Postpartum PREPS-Positive Appraisal

Variables	B	Lower CI	Upper CI	p-value
Indigenous (not Indigenous)***	1.11	0.37	1.84	<0.001
Racialized (not racialized)	0.02	-0.68	0.73	0.95
Immigrant (Canadian)	-0.20	-1.01	0.61	0.62
Education -Some Highschool/Highschool (post-graduate)	-0.03	-0.79	0.73	0.94
Education- College (post-graduate)	0.17	-0.29	0.62	0.47
Education- University (post-graduate)	0.14	-0.26	0.54	0.50
Income <$50,000 (>$150,000)	0.32	-0.43	1.07	0.40
Income $50,000 - $79,999 (>$150,000)	0.23	-0.39	0.85	0.47
Income $80,000 - $99,999 (>$150,000)	0.05	-0.46	0.55	0.86
Income $100,000 - $149,999 (>$150,000)	-0.08	-0.46	0.30	0.70
Age	-0.03	-0.07	0.01	0.19

Goodness of Fit: deviance/df=1.12 (df=166); Likelihood ratio $\chi^2(11)=16.06$; p=0.139 variables shown did not meet threshold for inclusion in final model. ***p<0.001. Reference categories shown in brackets. CI- 95% Confidence Interval

Table S21. Preliminary GLM: Healthcare Characteristics and Postpartum PREPS -Positive Appraisal

Variables	B	Lower CI	Upper CI	p-value
Primigravid (multigravid)	0.07	-0.23	0.37	0.65
Vaginal Delivery (C-section)	0.20	-0.14	0.53	0.25
HCP Ob/Gyn (midwife)	0.04	-0.46	0.53	0.88
HCP Family Physician (midwife)	-0.52	-1.24	0.19	0.15
Birthplace of Choice (no choice)	0.19	-0.33	0.71	0.47
Satisfied Prenatal Care Provider (not satisfied)	0.45	-0.14	1.03	0.13
Support Companion Restricted (not restricted)*	0.73	0.07	1.40	0.03
Prenatal Care Province-West (Atlantic)	-0.05	-0.69	0.59	0.88
Prenatal Care Province-Prairies (Atlantic)	-0.28	-0.82	0.25	0.29
Prenatal Care Province-Ontario (Atlantic)	0.25	-0.18	0.68	0.26
Prenatal Care Province-Quebec (Atlantic)	-0.22	-0.93	0.48	0.54
COVID Hot-Spot (low-no COVID)	0.40	-0.06	0.86	0.09
COVID Moderate (low-no COVID)	0.08	-0.37	0.53	0.73

Goodness of Fit: deviance/df=1.10 (df=166); Likelihood ratio $\chi^2(13)=22.50$; p=0.048. *p<0.05. Reference categories shown in brackets. CI- 95% Confidence Interval

Table S22. Preliminary GLM: Social Support, Coping and Postpartum PREPS-Positive Appraisal

Variables	B	Lower CI	Upper CI	p-value
OSS-3	0.01	-0.05	0.06	0.82
COPE-Problem-Based Coping Subscale*	0.40	0.09	0.71	0.01
COPE-Emotion-Based Coping Subscale	0.22	-0.23	0.66	0.34
COPE- Avoidant Based Coping Subscale	-0.16	-0.62	0.30	0.50

Goodness of Fit: deviance/df=1.09 (df=183); Likelihood ratio $\chi^2(4)=13.70$; p=0.008. *p<0.05. Reference categories shown in brackets. CI- 95% Confidence Interval
